# Physical Activity, Sedentary Behavior, Fruit and Vegetable Consumption, and Sarcopenia in Older Chinese Adults: A Cross-Sectional Study

**DOI:** 10.3390/nu15153417

**Published:** 2023-08-01

**Authors:** Yanjie Zhang, Xiaolei Liu, Yongzhi Ma, Xinxing Li

**Affiliations:** 1 Physical Education Unit, School of Humanities and Social Science, The Chinese University of Hong Kong, Shenzhen, Shenzhen 518172, China; zhangyanjie@cuhk.edu.cn; 2 Chinese Traditional Regimen Exercise Intervention Research Center, Beijing Sport University, Beijing 100084, China; liuxiaolei000@bsu.edu.cn; 3 Martial Arts Culture Research Center, Tsinghua University, Beijing 100084, China; myz@tsinghua.edu.cn; 4 Department of Physical Education, Seoul National University, Seoul 08826, Republic of Korea

**Keywords:** physical activity, sedentary, nutrition, sarcopenia

## Abstract

Purpose: The purpose of this study was to investigate the association between moderate to vigorous physical activity (MVPA), sedentary behavior, fruit and vegetable intake, and the risk of sarcopenia among older Chinese adults. Methods: This cross-sectional study enrolled 5418 older Chinese adults who participated in the Study on Global Aging and Adult Health (SAGE). Participants reported information about their physical activity, sedentary behavior, and dietary habits (fruit and vegetable intake). Sarcopenia was defined as the presence of low skeletal muscle mass and either a slow gait speed or weak handgrip strength. A multiple logistic regression model was employed to determine the relationship between MVPA, sedentary behavior, fruit and vegetable intake, and the risk of sarcopenia. Results: Only 32.63% of participants met all three recommendations (for MVPA, sedentary behavior, and fruit and vegetable intake). Compared with meeting none of the recommendations, meeting all three recommendations was associated with a lower risk of sarcopenia (OR = 0.63, 95% CI = 0.41–0.98). Moreover, meeting the recommendation for sufficient fruit and vegetable intake (OR = 0.69, 95% CI = 0.58–0.83), MVPA and fruit/vegetable intake (OR = 0.67, 95% CI = 0.52–0.86), and sedentary behavior and fruit/vegetable intake (OR = 0.69, 95% CI = 0.48–0.98) was associated with a lower risk of sarcopenia. Conclusion: Our findings indicate that in this large representative sample of older Chinese adults, meeting lifestyle recommendations for MVPA, sedentary behavior, and fruit and vegetable intake protected against sarcopenia.

## 1. Introduction

Throughout the history of human civilization, the yearning for eternal life has been a ubiquitous pursuit among humanity. As living conditions improve and medical technology continues to advance, there has been a significant increase in human life expectancy [1]. China has experienced remarkable demographic changes over the past few decades, which have been characterized by an unprecedented rate of aging [2]. According to Statistics in China, the number of individuals aged 60 and older could reach 300 million by 2025, and the rate of aging is projected to exceed 35% by 2050 [3]. This major demographic change poses serious challenges to the economy and healthcare system because age-related conditions, such as sarcopenia, impose considerable finical and psychological burdens on individuals, families, and society [4]. Sarcopenia is an age-related musculoskeletal disorder that is characterized by a progressive loss of skeletal muscle mass, strength, and function [5,6,7]. It is associated with various adverse outcomes, including physical disability, poor quality of life, an increased risk of falls and fractures, and higher healthcare costs [8,9,10]. For example, preoperative older adults with sarcopenia incur hospital costs of approximately $14,322 higher than their counterparts without this condition [11]. A study from Italy found that during the two years of the follow-up period, individuals with sarcopenia were more than three times as likely to experience falls compared to those without sarcopenia [12]. Moreover, recent studies have reported that sarcopenia is associated with an increased risk of cognitive impairment [11,13], depression [14], and unsatisfactory quality of life [15,16]. As the Chinese population continues to age, the prevalence of sarcopenia is expected to increase [17,18]. Therefore, it is important to recognize the risk factors and implement preventive strategies.

Previous studies have identified several factors as contributors to sarcopenia, including hormonal changes, chronic inflammation, and lifestyle behaviors [16,19]. Among lifestyle behaviors, physical activity, sedentary behavior, and dietary habits have emerged as key modifiable factors that could influence the development and progression of sarcopenia [20,21,22,23]. Physical activity is defined as any form of bodily movement generated by skeletal muscles, entailing energy expenditure [24]. From the perspective of preventative medicine, regular physical activity, especially moderate to vigorous physical activity (MVPA), is an essential health behavior and has been consistently linked to the preservation of muscle mass, strength, and function in older adults. For example, observational epidemiologic studies have investigated the association between physical activity and skeletal muscle hypertrophy, positive physical performance, and sarcopenia [25,26]. In a systematic review including 17 randomized controlled studies, engaging in physical activity, especially resistance training, stimulates muscle protein synthesis and promotes hypertrophy [7].

Sedentary behavior is defined as an activity performed in a sitting, lying, or reclining position while awake, with an energy expenditure of less than 1.5 metabolic equivalents (METs). Sedentary behavior is associated with accelerated muscle loss, functional decline, and sarcopenia [15,27]. This may be because sedentary behavior is related to elevated levels of deep adipose tissue and visceral fat, which have a catabolic effect on the muscle by promoting protein degradation [28,29]. For example, Hamer et al. found a significant relationship between long sitting times (e.g., watching TV) and lower muscle strength in older adults [30]. Smith et al. reported that high levels of sedentary behavior are associated with confirmed sarcopenia among residents of low- and middle-income countries [28]. Moreover, Gianoudis et al., in their cross-sectional study with 162 community-living older males and females, found that for every additional hour of sitting time, the risk of sarcopenia was increased markedly by 33% [31].

Moreover, dietary habits also play a crucial role in muscle health maintenance. Adequate nutrition, such as the consumption of high-quality protein, vitamin C, and vitamin D, is essential for muscle protein synthesis and repair [32,33]. Additionally, fruits and vegetables are rich in antioxidants and bioactive compounds, and they possess anti-inflammatory and antioxidant properties that can benefit muscle health [34,35]. Indeed, Hashemi et al.’s cross-sectional study with older Iranian adults found that high consumption of fruits and vegetables was associated with a reduced likelihood of muscle dystrophy [36]. Similarly, a cross-sectional study conducted in South Korea reported a positive association between fruit and vegetable intake and sarcopenia in older men [37]. Furthermore, a randomized controlled study conducted in the United Kingdom showed that older individuals who ate more fruits and vegetables had a greater tendency to increase their handgrip strength [38]. However, the relationship between fruit and vegetable intake and sarcopenia in older Chinese adults remains unclear.

Although researchers have explored the relationships between physical activity, sedentary behavior, dietary habits, and sarcopenia, few studies have investigated the combined effects of these lifestyle factors [15]. Examining the interplay between MVPA, sedentary behavior, and fruit and vegetable intake could provide valuable insights into their independent and synergistic contributions to sarcopenia risk. Therefore, the purpose of this study was to examine the relationship between MVPA, sedentary behavior, fruit and vegetable intake, and the risk of sarcopenia in older Chinese adults.

## 2. Methods

### 2.1. Participants

This study utilized data from the Study on Global Ageing and Adult Health (SAGE) to examine the relationship between sarcopenia risk and meeting recommendations for physical activity, sedentary behavior, and fruit and vegetable intake in older Chinese adults. The SAGE survey employed a stratified multistage cluster sampling design, which ensured that diverse geographical locations and economic statuses were represented. The sample consisted of individuals aged 65 years and older from eight provinces in China: Guangdong, Hubei, Jilin, Shaanxi, Shandong, Shanghai, Yunnan, and Zhejiang. In total, 5418 participants were included in the analysis after eliminating missing data.

The data collection process followed a standardized approach, as described in a previous study [39]. Well-trained interviewers provided clear instructions to participants on how to complete the questionnaire accurately. The survey achieved a high response rate of 93%. This study adhered to the ethical considerations of the Declaration of Helsinki. The study obtained ethical approval from the Ethics Review Committee of Chinese Center for Disease Control and Prevention and the WHO Ethical Review Committee. All participants provided informed consent by agreeing to participate and signing a consent form [40].

### 2.2. Physical Activity

The Global Physical Activity Questionnaire (GPAQ, version 2) was employed to assess physical activity [41]. This instrument assesses the frequency (the days in a typical week) and duration (hours and minutes in a typical day) of moderate and vigorous physical activity conducted in three domains (work, travel, and recreation). Physical activity was computed from the three physical activity domains and converted to METs in minutes (MET-min) per week. The WHO classifies insufficient physical activity as 0–600 MET min per week, moderate physical activity as 601–3000 MET min per week, and high physical activity as over 3000 MET min per week [42]. Adequate MVPA was defined as meeting the physical activity recommendation [42].

### 2.3. Sedentary Behavior

Sedentary behavior was measured by the following questionnaire item: “How much time do you usually spend sitting or reclining on a typical day? Such as sitting at a desk, sitting with friends, traveling in a car, bus, train, reading, playing cards, or watching television” [34]. This question was taken from the GPAQ. In this study, sedentary behavior was assessed with both a continuous and a categorical variable. The continuous variable was the total number of hours per day that participants reported spending in sedentary activities. The categorical variable divided participants into groups according to their reported sedentary behavior time. The categories were <8 h per day and ≥8 h per day.

### 2.4. Fruit and Vegetable

Participants were asked the two following questions: “How many servings of fruit do you eat on a typical day?” and “How many servings of vegetables do you eat on a typical day?” [43] The participants were grouped into two categories (Yes or No) according to their answers to these questions. For fruit intake, ‘yes’ was defined as eating two or more servings of fruit a day, and ‘no’ was defined as eating less than two servings. For vegetable intake, ‘yes’ was defined as eating three or more servings of vegetables a day, and ‘no’ was defined as eating less than three servings [44].

### 2.5. Sarcopneia

Following the criteria outlined in the revised European consensus on the definition and diagnosis of sarcopenia [6], sarcopenia was identified by low skeletal muscle mass (SMM), as defined by a low skeletal muscle mass index (SMI) and weak handgrip strength. SMM was calculated with the equation proposed by Lee et al. [45], which includes weight, height, sex, age, and race. The equation is as follows: SMM = 0.244 × weight + 7.8 × height + 6.6 × sex − 0.098 × age + race − 3.3. For sex, a value of 0 represents female, and a value of 1 represents male. For race, a value of −1.2 is used to represent people of Asian nationality. SMM was divided by body mass index (BMI) to generate the SMI. The lowest quantile of SMI based on sex-specific values was considered to indicate low SMM. Handgrip strength was assessed as an indicator of muscle strength. Handgrip strength was calculated as the mean value of two handgrip measurements on the dominant hand. Weak handgrip strength was defined as less than 28 kg for men and less than 18 kg for women [5]. Gait speed, measured by a 4 m timed walk, was used as a measure of physical performance. Participants were instructed to walk at their normal pace, and the time to complete the 4 m walk was recorded. A gait speed of 0.8 m/s or less was considered a slow gait speed [40].

### 2.6. Control Variables

The control variables were age, gender, education (less than elementary school, elementary school, middle school or high school, and college or post-graduate degree), the number of diseases (e.g., diabetes, hypertension, arthritis, stroke, etc.), residential area (rural and urban), smoking (current, past, and never smoking), alcohol consumption, and BMI. The number of chronic diseases was determined by summing the number of self-reported diagnosed conditions. BMI was computed as weight (in kilograms)/height^2^ (in meters).

### 2.7. Statistical Analysis

Data were expressed as counts, percentages, or means, and the standard deviation (SD) in this study. The multivariable logistic regression model was used to explore the association between sarcopenia risk and the number of recommendations met (e.g., the participant met none, one, two, or three of the recommendations for MVPA, sedentary behavior, and fruit and vegetable intake) and the association between sarcopenia risk and the specific combinations or recommendations met (none, MVPA only, sedentary behavior only, and fruit and vegetable intake only, MVPA + sedentary behavior, MVPA + fruit and vegetable intake, sedentary behavior + fruit and vegetable intake, or all three combined). All models were fully adjusted for covariates (e.g., age, sex, education, number of chronic diseases). The results are presented as the odds rates (ORs) with 95% confidence intervals (CIs). The statistical software Stata 15.0 (Stata Corp LP, College Station, TX, USA) was employed for the analysis, and statistical significance was defined as a two-sided *p*-value of *p* < 0.05.

## 3. Results

Table 1 presents the participants’ characteristics. The study included a total of 5418 adults aged over 65 years old. Approximately half of the participants were women (*n* = 2867, 52.92%). The mean age of participants was 72.92 (±5.86) years; 5.8% of participants had an education level of college or above; 45% of participants lived in rural areas; and 26.61% and 65.62% of participants had no history of alcohol consumption and smoking, respectively. The prevalence of sarcopenia was 15.17%. Only 32.63% of participants met all three recommendations, and 3.48% of participants did not meet any of the recommendations.

**Figure 1:**
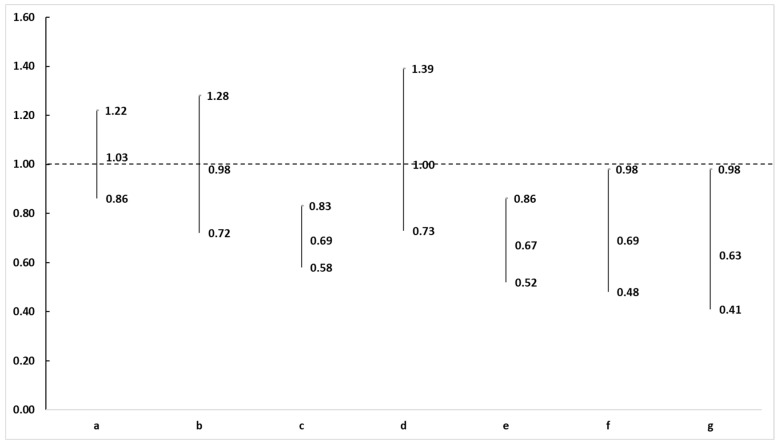
Associations of meeting guidelines or recommendations with sarcopenia: ORs and 95% CIs. ORs, odds rates; CIs, confidence intervals; a: Meeting moderate-to-vigorous physical activity recommendation; b: Meeting sedentary (less than 8-h/day) recommendation; c: Meeting fruit and vegetable consumption recommendation; d: Meeting moderate-to-vigorous physical activity and sedentary recommendations; e: Meeting moderate-to-vigorous physical activity and fruit and vegetable consumption recommendations; f: Meeting sedentary and fruit and vegetable consumption recommendations; g: Meeting all three recommendations. The dashed line represents 1, it means that there is no significant association between the meeting recommendations and sarcopenia risk.

As shown in Table 2 and Figure 1, all three recommendations were associated with sarcopenia risk in the unadjusted model. Specifically, participants with high MVPA, low sedentary behavior (<8 h), and sufficient fruit and vegetable intake had a lower risk of sarcopenia. After controlling for all covariates, the strengths of most relationships decreased. However, reduced sarcopenia risk was still associated with meeting all three recommendations (OR = 0.63, 95% CI = 0.41–0.98). In addition, the results indicated that meeting one or two combinations could protect against sarcopenia. Specifically, sufficient fruit and vegetable intake alone (OR = 0.69, 95% CI = 0.58–0.83), both high active MVPA + fruit and vegetable intake (OR = 0.67, 95% CI = 0.52–0.86) and low sedentary behavior + fruit and vegetable intake (OR = 0.69, 95% CI = 0.48–0.98), were associated with a lower risk of sarcopenia.

## 4. Discussion

To our knowledge, this is the first study to investigate the relationship between lifestyle recommendations (MVPA, sedentary behavior, and fruit and vegetable intake) and sarcopenia risk in older Chinese adults. This study included a large sample size of 5418 participants aged over 65 years; approximately half of the participants were women, and the mean age was 72.92 years. Our results indicate that meeting the fruit and vegetable intake recommendation alone, meeting both MVPA and fruit and vegetable intake recommendations, meeting both the sedentary behavior and fruit and vegetable intake, and meeting all three recommendations were significantly associated with lower sarcopenia risk.

Previous studies have reported that nutrition (e.g., protein intake, omega-3 fatty acids, and fruit and vegetable intake) is an important factor in improving muscle strength and function in older adults [46,47]. Our findings are in line with the results of a previous study conducted by Kim et al. [37], which found that older adults with adequate fruit and vegetable consumption had a lower risk of sarcopenia compared with those with inadequate fruit and vegetable consumption. This is because fruits and vegetables are rich in nutrients that are important for muscle health, including vitamins C and E, beta-carotene, and potassium [48,49]. These antioxidant nutrients can help reduce oxidative stress and inflammation, which are known risk factors for sarcopenia [50,51]. For instance, Saito et al. reported a closed relationship between low plasma vitamin C levels and poor physical function in older adults [52]. Moreover, Cesari et al. found that high antioxidant intake (i.e., vitamin C and beta-carotene) was associated with greater muscle strength in older adults [53]. Therefore, consuming a diet that is rich in fruits and vegetables could be a useful strategy to prevent or mitigate the development of sarcopenia in older Chinese adults.

Meeting lifestyle behavior recommendations is associated with superior physical health [54,55] and is linked to a lower risk of having sarcopenia [56]. The current study is the first to demonstrate that meeting the recommendations for both MVPA and fruit and vegetable intake, meeting the recommendations for both sedentary behavior and fruit and vegetable intake, and meeting all three recommendations can reduce the risk of sarcopenia in older Chinese adults. Our findings also indicate that the risk of sarcopenia decreases as the number of recommendations met increases. Meeting all three recommendations for MVPA, including sedentary behavior, and fruit and vegetable intake, is the best combination for preventing sarcopenia. Similarly, another study found that meeting all three recommendations was more likely to improve muscle growth and repair [54]. In addition, previous research has indicated that meeting all three recommendations prevents disability in older adults; individuals with recommended levels of physical activity, sedentary behavior, and fruit and vegetable intake effectively tend to exhibit superior physical performance compared with those who do not meet the recommendations [57,58]. A combination of physical activity, limited sedentary behavior and a nutritious diet has synergistic effects and creates an optimal environment for muscle synthesis, preservation, and repair, leading to a decreased risk of sarcopenia. Hence, older adults who meet the recommendations for MVPA, sedentary behavior, and fruit and vegetable intake may have a lower risk of developing sarcopenia.

Individuals who meet the recommendations for both MVPA and fruit and vegetable intake, as well as those who meet the recommendations for both sedentary behavior and fruit and vegetable intake, may have a decreased risk of sarcopenia. The present study indicated that meeting the fruit and vegetable intake recommendation was protective against sarcopenia, and sufficient MVPA and limited sedentary behavior strengthened this relationship. Physical activity has long been recognized as a key factor in maintaining muscle and physical performance. Participating in regular exercise, such as resistance training and aerobic activities, can stimulate muscle protein synthesis, increase insulin sensitivity, and trigger the release of growth factors that support muscle growth and repair [57]. By contrast, sedentary behavior limits muscle contractile stimulation, energy expenditure, and muscle metabolism, leading to an imbalance between protein synthesis and breakdown [31,59]. Therefore, limiting sedentary behavior can improve muscle synthesis and growth, contributing to a lower risk of sarcopenia [59]. Moreover, adequate fruit and vegetable intake can have a synergistic effect that promotes muscle and physical performance in older adults [57,58]. Each recommendation targets different aspects of muscle function and physical performance and provides complementary benefits [23]. Therefore, older adults should be encouraged to meet two or more healthy lifestyle behavior recommendations to reduce their risk of sarcopenia. However, more research is required to verify the results of the present study.

Our observations may have important implications for the aging population in China. These findings highlight the importance of a healthy lifestyle (engaging in physical activity, limiting sedentary behavior, and consuming fruits and vegetables) in preventing sarcopenia in older adults. Health experts should consider encouraging older adults to engage in physical activity, limit sedentary behavior, and consume sufficient amounts of fruits and vegetables during nutritional consultations and education. Moreover, policymakers should recommend fruit and vegetable consumption and build exercise-friendly communities to improve the health status of older adults and prevent the incidence of sarcopenia in China.

The present study extended the existing evidence in this field by assessing physical activity, sedentary behavior, and fruit and vegetable intake in a large and representative population of older Chinese adults. Moreover, we used adjusted models that controlled for a series of covariates to reduce a confounding bias. Nonetheless, some limitations should be acknowledged in our study. First, the study utilized a cross-sectional design and, thus, could not establish a causal relationship between a healthy lifestyle and sarcopenia risk. Second, we could not determine that there was no inverse causality; for example, it was challenging to ascertain whether individuals adopted certain behaviors due to their health status [31], although various lifestyle factors and potential confounders (e.g., the history of chronic diseases) were taken into consideration in the analyses. Third, all measurements relied on self-reported questionnaires or indirect estimates of skeletal muscle mass, which could be affected by recall bias and over- or under-estimations. Therefore, future studies should consider using objective measures to validate self-reported measures to verify our findings. Finally, because this study was conducted in a Chinese population, the generalizability of the findings to other populations is limited.

## 5. Conclusions

In conclusion, the current study provides evidence that meeting lifestyle behavior recommendations for MVPA, sedentary behavior, and fruit and vegetable intake can reduce sarcopenia risk in older Chinese adults. Our findings suggest that adopting a healthy lifestyle may be an effective strategy to prevent sarcopenia in older adults, while future studies are still needed to confirm the association between sarcopenia risk and meeting recommendations for MVPA, sedentary behavior, and fruit and vegetable intake in older Chinese adults.

## Figures and Tables

**Table 1:** Sample characteristics.

Variables		*n*	Mean ± SD/%
Age		5418	72.92 ± 5.86
Gender	Male	2551	47.08
	Female	2867	52.92
Education (years)	0	862	15.91
	1–6	1555	28.71
	7–12	509	9.39
	>12	314	5.80
	Missing	2178	40.20
Residential areas	Rural	2441	45.05
	Urban	2977	54.95
Alcohol consumption	Yes	1442	26.61
	No	3758	69.36
	Missing	218	4.03
Smoking	Never	3556	65.62
	Current	1178	21.74
	Past	465	8.58
	Missing	209	3.86
Number of chronic diseases	0	1926	35.55
	1–2	2599	47.97
	≥3	572	10.56
	Missing	321	5.92
Sarcopenia (yes)		822	15.17
	Missing	613	11.31
Recommendations met			
None		169	3.48
MVPA		3133	57.83
Sedentary behavior		4890	90.25
Fruit and vegetable intake		2877	53.10
MVPA + sedentary hehavior		2953	54.50
MVPA + fruit and vegetable intake		1865	34.42
Sedentary behavior + fruit and vegetable intake		2625	48.45
MVPA + sedentary + fruit and vegetable intake		1768	32.63

Note: MVPA, moderate-vigorous physical activity; SD, standard deviation.

**Table 2:** Associations between MVPA, sedentary behavior, fruit and vegetable intake, and sarcopenia risk.

	Unadjusted				Adjusted ^#^			
	*p*	OR	95%CI		*p*	OR	95%CI	
Specific combinations								
None (reference)	-	-	-	-	-	-	-	-
MVPA only	<0.001	0.68	0.59	0.79	0.77	1.03	0.86	1.22
Sedentary behavior only	<0.01	1.35	1.08	1.70	0.90	0.98	0.75	1.28
Fruit and vegetable intake only	<0.001	0.49	0.41	0.57	<0.001	0.69	0.58	0.83
MVPA + sedentary behavior	<0.001	0.53	0.40	0.70	0.99	1.00	0.73	1.39
MVPA + fruit and vegetable intake	<0.001	0.35	0.28	0.43	<0.01	0.67	0.52	0.86
Sedentary behavior + fruit and vegetable intake	<0.001	0.34	0.25	0.47	0.04	0.69	0.48	0.98
All	<0.001	0.26	0.18	0.37	0.04	0.63	0.41	0.98
General combinations								
None (reference)	-	-	-	-	-	-	-	-
One	<0.01	0.57	0.40	0.82	0.12	0.72	0.48	1.09
Two	<0.001	0.35	0.25	0.49	<0.01	0.61	0.41	0.90
Three	<0.001	0.26	0.18	0.37	<0.01	0.63	0.41	0.98

Note: OR odds ratio; CI confidence interval; MVPA, moderate-vigorous physical activity. ^#^ The models are adjusted for age, gender, education years, residential areas, alcohol consumption, smoking, number of chronic diseases, and all the other characteristics in the table.

## Data Availability

The datasets for this study can be requested from the authors.
